# Predictive modelling of vascular surgery trends using machine learning: a comparative study of Irish public and private tertiary referral centres

**DOI:** 10.3389/fsurg.2025.1733205

**Published:** 2026-01-08

**Authors:** Sherif Sultan, Yogesh Acharya, Mohamed S. Sultan, Omnia Zayed, Osama Soliman

**Affiliations:** 1 Department of Vascular and Endovascular Surgery, Western Vascular Institute, University Hospital Galway, University of Galway, Galway, Ireland; 2 Department of Vascular and Endovascular Surgery, Galway Clinic, Royal College of Surgeons in Ireland and the University of Galway Affiliated Teaching Hospitals, Galway, Ireland; 3 Royal College of Surgeons in Ireland (RCSI), Dublin, Ireland; 4 Letterkenny University Hospital, Letterkenny, Ireland; 5 Insight Research Ireland Center for Data Analytics, University of Galway, Galway, Ireland; 6 Cardiovascular Research Institute (CVRI) & PRECISE Core Labs, Mater Private Network, Dublin, Ireland; 7 The Euro Heart Foundation, Rotterdam, Netherlands

**Keywords:** vascular surgery, predictive models, machine learning, public-private hospitals, trend analysis

## Abstract

**Background:**

Vascular diseases are increasing in Ireland as well as worldwide alongside an ageing society, posing a growing demand for trained and qualified healthcare professionals. In this study, we have analysed current practices of vascular interventions by using the data from the vascular tertiary centre to predict the future size and capacity of the vascular surgery workforce through artificial intelligence (AI)-powered predictive models.

**Methods:**

We employed supervised machine learning (ML) regression model to predict trends in the landscape of complex vascular and endovascular surgery over the next 22 years, utilising data from a high-volume public and private tertiary referral vascular centre spanning two decades (2002 to 2023) in the West of Ireland.

**Results:**

We conducted 1,653 aortic interventions, 1,185 carotid interventions, and 3,069 peripheral vascular interventions, with conversion rates from referral to surgery of 5%, 7.4%, and 9.8%, respectively. The private sector experienced a dramatic 73-fold increase in abdominal aortic aneurysm interventions, contrasted with a modest 1.25-fold increase in the public sector. Our model predicts a shortage of vascular surgeons, with the workforce potentially meeting demand by 2050. By 2030, each surgeon would need to increase yearly wRVU production by 22%–31% and by 2040 by 8%–11% to accommodate the workload.

**Conclusions:**

Our model predicts a shortage of the vascular surgery workforce over the next two decades. We can speculate that addressing future needs in vascular surgery requires either training more specialists or increasing the efficiency and wRUV through strategic planning and integration of AI/ML systems to ensure adequate compensation and the sustainability of the workforce. By focusing on these areas, we can navigate the evolving landscape of vascular surgery and continue providing high-quality patient care.

## Introduction

Over the past three decades, vascular surgery has undergone a remarkable transformation, marked by significant shifts in patient presentation, management strategies, and healthcare disparities. Advancements in medical technology and changes in healthcare delivery systems have influenced the understanding and treatment of vascular diseases (1–3). A notable disparity in the types of vascular interventions between patients with private insurance and those from deprived social backgrounds underscores the need for equitable access to comprehensive vascular care (4, 5). Additionally, the private sector has substantially increased the number of vascular interventions, raising questions about healthcare equity and resource allocation (5).

Early intervention in vascular diseases significantly impacts patient outcomes and healthcare costs. However, the coronavirus disease 2019 (COVID-19) pandemic has introduced unprecedented challenges, highlighting the need for a sustainable vascular surgery workforce (6–8). It is important to study the trends in surgical practice and workforce adequacy for workforce planning. ML models, compared to the conventional regression, can capture complex data interactions and non-linear patterns often seen in real-world clinical data (9, 10). Furthermore, they also improve predictive accuracy and helps in automatic feature learning, especially in complex data sets. Therefore, we have analysed current practices of vascular interventions to predict the future size and capacity of the vascular surgery workforce through artificial intelligence (AI) - powered predictive models, highlighting concerns about its current and future adequacy.

## Methods

We created a comprehensive database of patients who have undergone various vascular surgical procedures over 22 years (2002–2023) at our tertiary referral centre —public hospital (PUB) and private practice (PRV) in the West of Ireland. Ethical approval for aortic, carotid, and peripheral vascular disease across the two institutes, both high-volume public and private tertiary-level vascular referral centres, was obtained from the institutional clinical research ethics committee (approval IDs: C.A.2635, C.A.2636, C.A.2637, and CA190520). All procedures were conducted in accordance with relevant guidelines and regulations.

**Figure 1:**
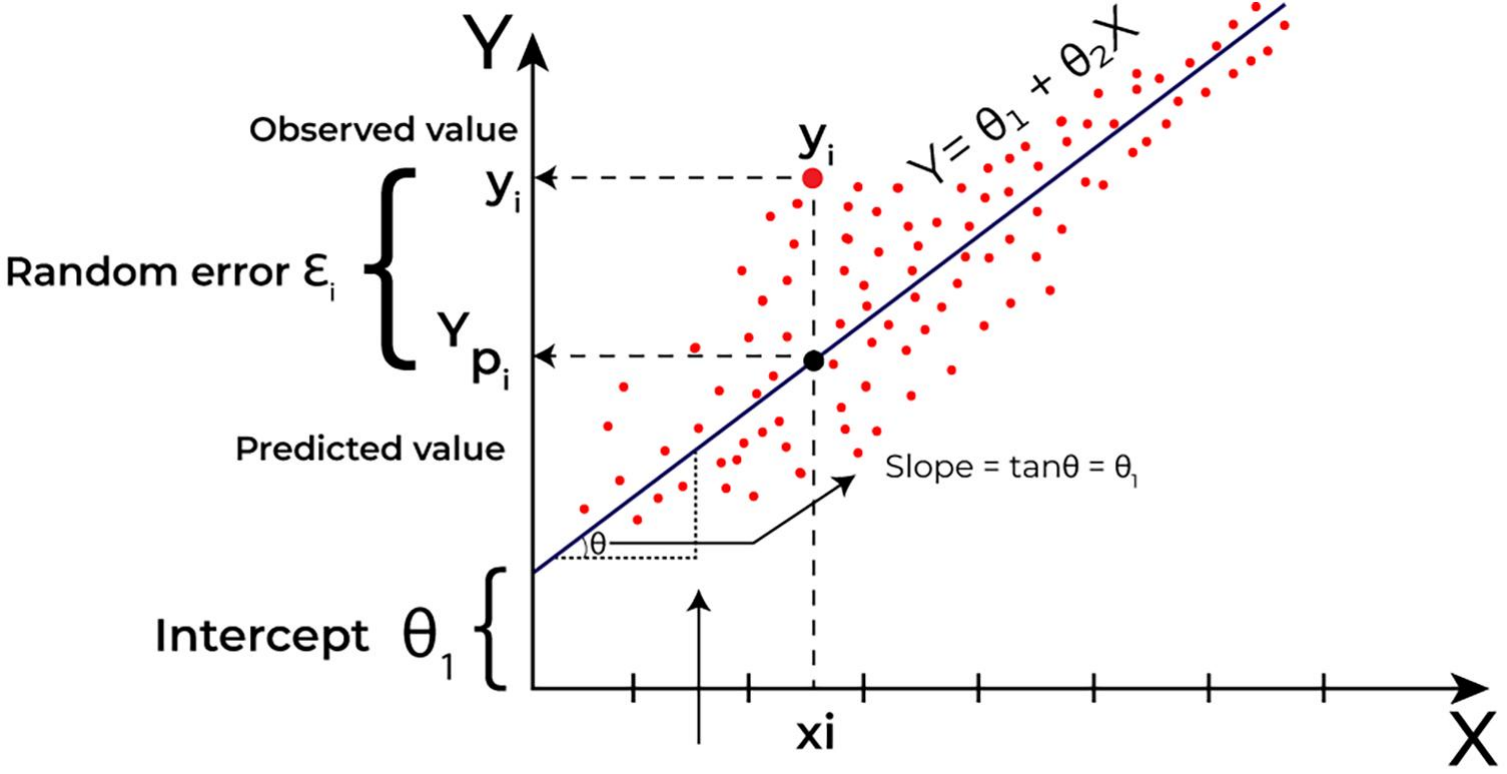
Modelling the predicted workforce of vascular surgery in Ireland by the year 2050.

Demographic and perioperative data were evaluated, and patients were categorized by surgical procedure and the relevant cause. Updated population estimates were obtained from the Irish Census Bureau. A subset of regression-based supervised machine learning (ML) applications was used to forecast workforce requirements, and model accuracy was assessed using R-squared values. Forecasting used the nearest buckets to improve accuracy, noting that predictions for 2023 are more accurate than those for 2050 (Figure 1).

### Data processing and preparation

Data were obtained from a secure clinical database containing vascular surgery records from public and private tertiary referral centres in Galway, Ireland. The dataset included procedures classified into aortic, carotid, and peripheral vascular disease categories. Data collection began in 2002 and followed a structured, periodically audited process. The two contributing centres conducted formal reviews of the database approximately every four years to ensure accuracy, completeness, and internal consistency.

No statistical outliers were identified during the preliminary inspection, as procedure counts and patient-level variables had already been validated as part of the database's maintenance procedures. All data were re-examined, cleaned, and pre-processed prior to modelling. Data cleaning steps included verifying variable coding, confirming temporal consistency across years, and checking for missing or duplicate entries. Variables were standardised where necessary, and categorical variables were encoded appropriately. Because the dataset represented a complete census of available records for the study period, no imputation procedures were required. Finally, this prepared dataset was used directly for the regression-based supervised ML predictive modelling performed in the study

### Predictive modelling methods of vascular interventions over time

We used ML-based regression analysis as a statistical method to relate the number of surgeries to one or more independent variables. Several modelling methods were employed, including linear Regression, Polynomial Regression, Power Regression, and Exponential Modelling.

#### Linear regression method

This method assumes a straight-line relationship between the dependent variable (e.g., number of interventions) and the independent variable (e.g., time). It's used when the data points exhibit a linear trend. For example, if the number of interventions increases constantly over time, linear regression provides a simple yet effective predictive model.

#### Polynomial regression

In polynomial regression, the relationship between the independent and dependent variables is modelled as an *n*^th^-degree polynomial. This method is suitable for data that shows a curvilinear trend, meaning the rate of increase or decrease changes over time. For instance, if the number of interventions grows at an accelerating or decelerating rate, polynomial regression captures this non-linear pattern.

#### Power regression

Power regression models describe relationships where the dependent variable changes as a power of the independent variable. This non-linear approach is used when the data suggests that interventions grow or shrink proportionally to the variable raised to a power. It's useful when the data follows a multiplicative rather than an additive pattern.

#### Exponential modelling

This type of modelling is ideal for datasets where values change exponentially, either increasing or decreasing at an accelerating rate. Exponential models are appropriate when the rate of change is proportional to the current value, indicating a constant relative rate of change. This is useful for predicting trends in which growth or decay speeds up over time, such as rapidly increasing intervention rates.

These models are essential tools for predictive analytics, enabling businesses, analysts, and healthcare professionals to forecast future trends, demand, and behaviour by leveraging historical data. Understanding and applying these techniques can lead to more accurate predictions and better decision-making.

### Use of observed data for predictive modelling

We used observed data from two deliberate practice volume vascular centres in the West of Ireland, both of which use the same triage, management, and technology trends. Examining trends and shifts in surgical volumes will provide a foundation for predictive modelling and future planning.

### Work force prediction

Our model uses the most recent Irish Census Bureau population projections, published in 2024, to estimate the number of vascular surgeons required to provide health care to the Irish population (11). Irish Medical Council Registered Specialists data for 2023 were used to identify the number of practising vascular surgeons in 2023. We used the data previously described by Go et al. (12), a ratio of 1.4 vascular surgeons per 100,000 population. This data was derived by Richard Cooper and used by Merritt Hawkins in “A market analysis of vascular surgery supply, demand, compensation and recruiting trends: prepared for the Society for Vascular Surgery, November 13, 2017” to estimate vascular surgery workload (12). Although not assumed to be directly transferable to the Irish system, we have used this United States - based benchmark as a pragmatic external reference in the absence of Ireland-specific vascular surgery workforce ratios. In addition, the median productivity of vascular surgeons, estimated at 8,481 work relative value units (wRVU)/year, was derived from the 2017 wRVU data from the “Medical Group Management Association Physician Compensation and Production” reports.

## Results

We conducted 1,653 aortic interventions, 1,185 carotid interventions, and 3,069 peripheral vascular interventions, with conversion rates from referral to surgery of 5%, 7.4%, and 9.8%, respectively. The private sector experienced a dramatic 73-fold increase in abdominal aortic aneurysm interventions, contrasted with a modest 1.25-fold increase in the public sector. Similarly, carotid surgeries surged 11.8-fold in the private sector, while the public sector saw a 0.69-fold decrease. Peripheral interventions showed a 3.36-fold increase in the private sector compared to a 0.87-fold decrease in the public sector. The trend towards endovascular or hybrid procedures has continued, with carotid endarterectomy remaining the gold standard surgical intervention.

We estimated the median productivity of vascular surgeons at 8,481 wRVUs per year and calculated excess or deficit in wRVU capacity. Our model predicts a shortage of vascular surgeons through 2040, with the workforce potentially meeting demand by 2050. By 2030, each surgeon would need to increase yearly wRVU production by 22%–31% and by 2040 by 8%–11% to accommodate the workload. The projected congruence between workforce and demand in 2050 may be related to increases in the number of trainees from integrated residencies and decreases in population estimates. Disparities in vascular interventions were noted between patients with private insurance and those from deprived backgrounds, with the latter more likely to present with advanced peripheral vascular disease and higher rates of amputations. Ireland has one of the highest rural populations in Europe, and nearly 2/3rd of the population reside in the rural areas in the West of Ireland (13). These rural populations have poor health literacy, low private health expenditure, and overall limited access to healthcare, including both public and private healthcare institutions (14, 15).

### Aortic surgeries absolute numbers (2002–2023)

**Figure 2:**
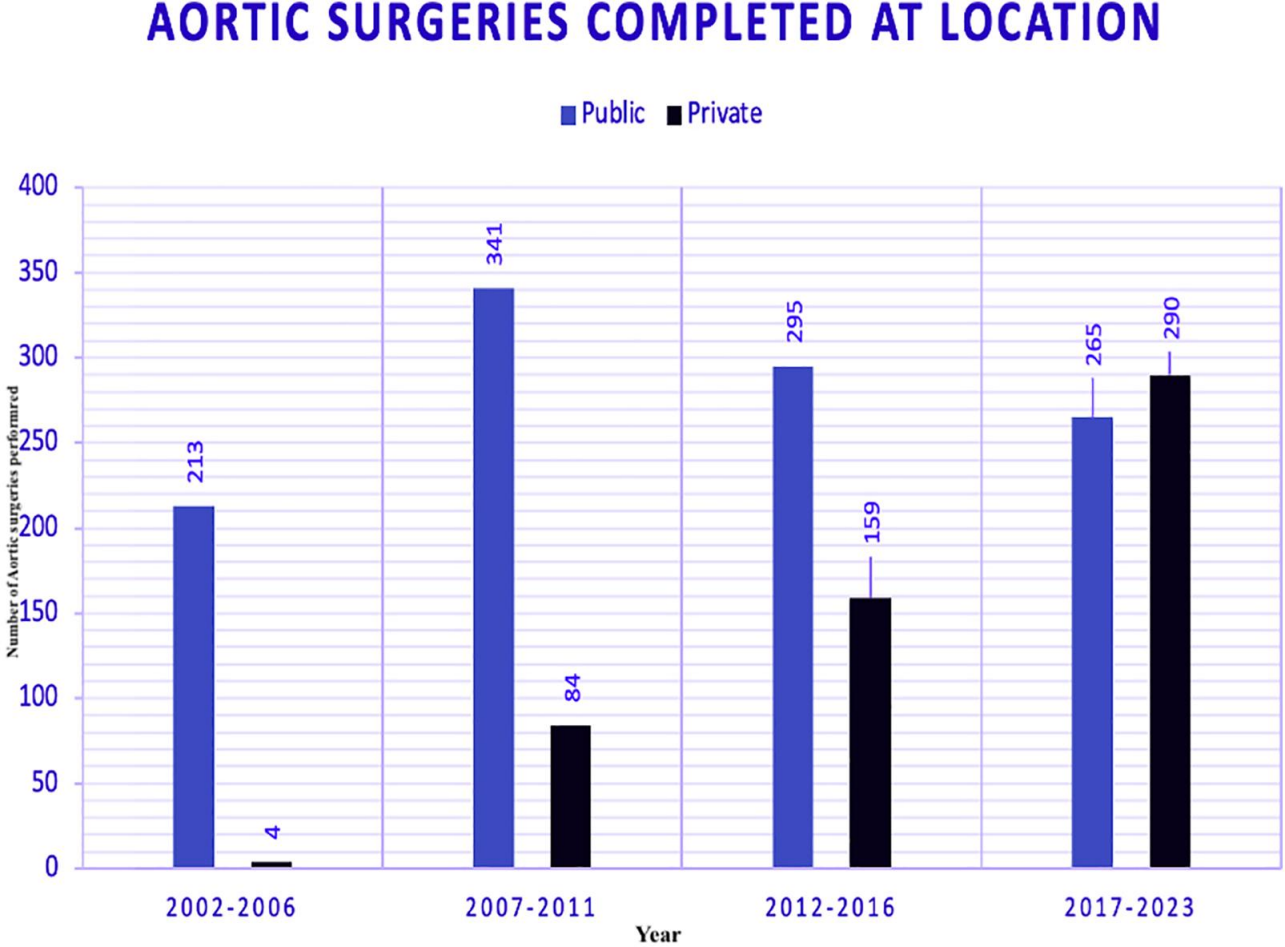
Aortic surgeries segmented by era and sector between public (PUB) and private (PRV) hospitals.

Data on aortic surgeries were segmented into four five-year eras, comparing public and private sectors (Figure 2). The number of surgeries has evolved in both public and private hospitals.

### Carotid surgeries absolute numbers (2002–2023)

**Figure 3:**
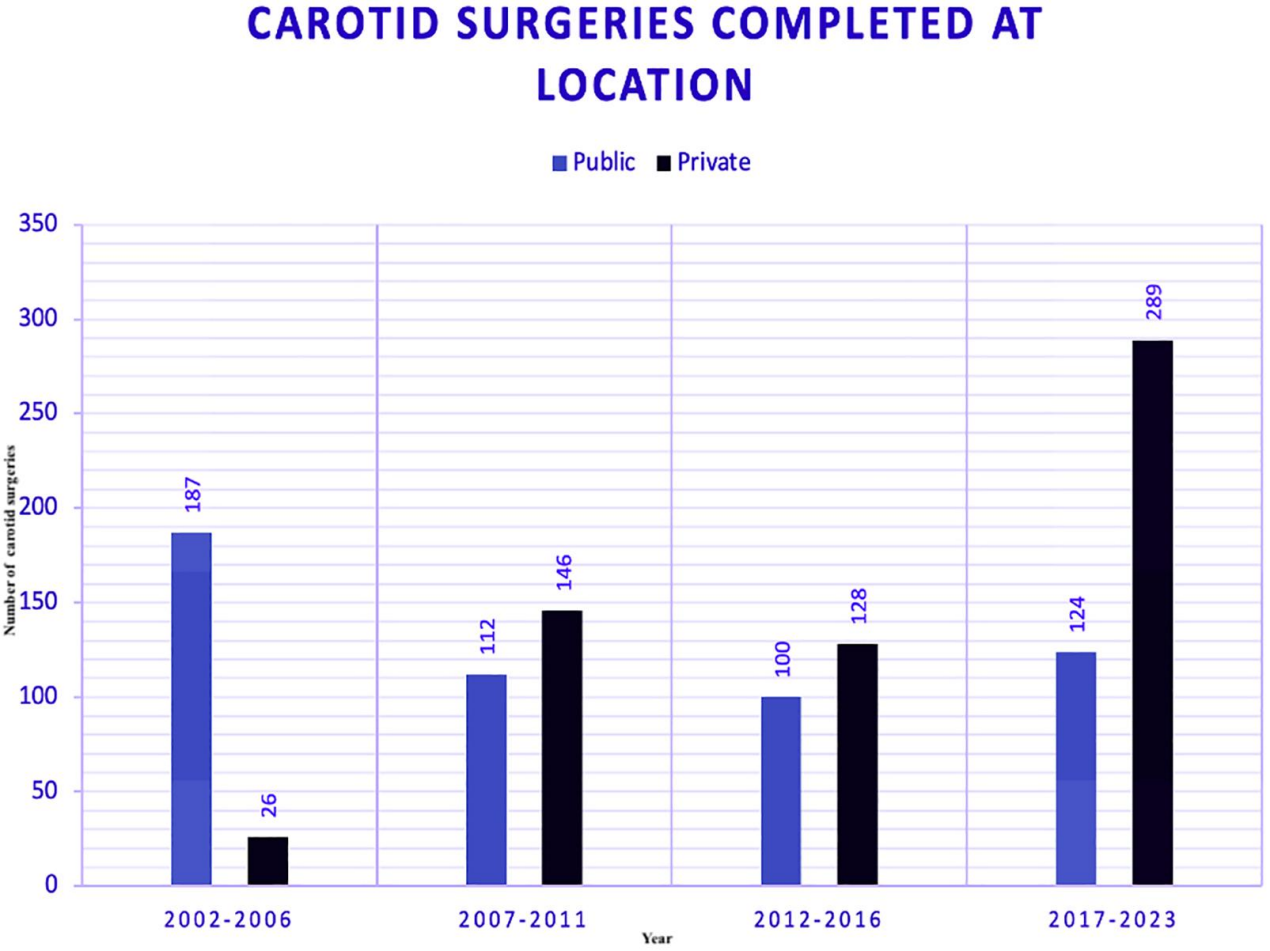
Carotid surgeries segmented by era and sector between public (PUB) and private (PRV) hospitals.

Data on aortic surgeries were segmented into four five-year eras, comparing the public and private sectors (Figure 3). This visualisation highlights the trends in surgical volumes over time in both public and private hospitals.

### Peripheral surgeries absolute numbers (2002–2023)

**Figure 4:**
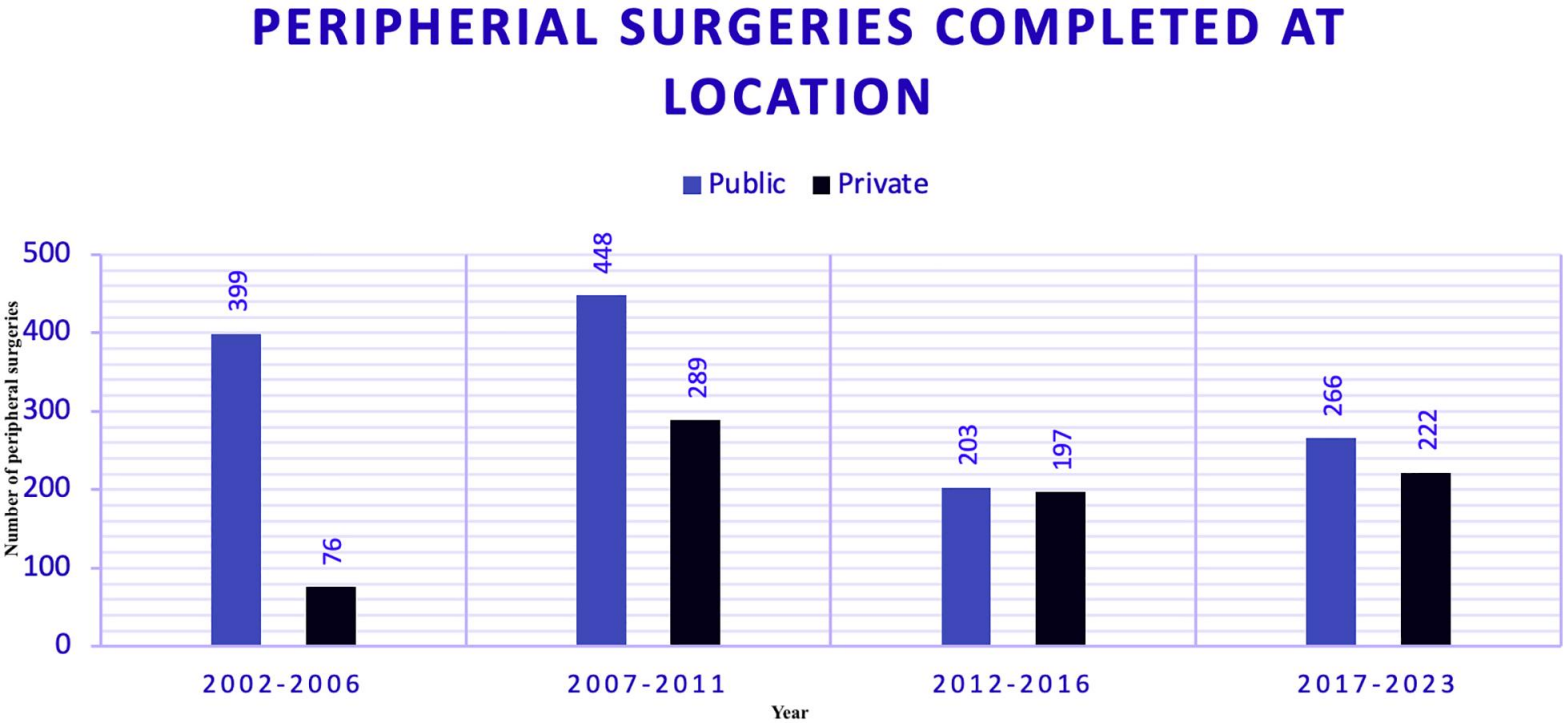
Peripheral surgeries segmented by era and sector between public (PUB) and private (PRV) hospitals.

Data on Carotid surgeries were segmented into four five-year eras, comparing the public and private sectors (Figure 4). This visualization highlights the trends in surgical volumes over time in both public and private hospitals.

### Predicted aortic surgeries trends (2002–2023)

**Figure 5:**
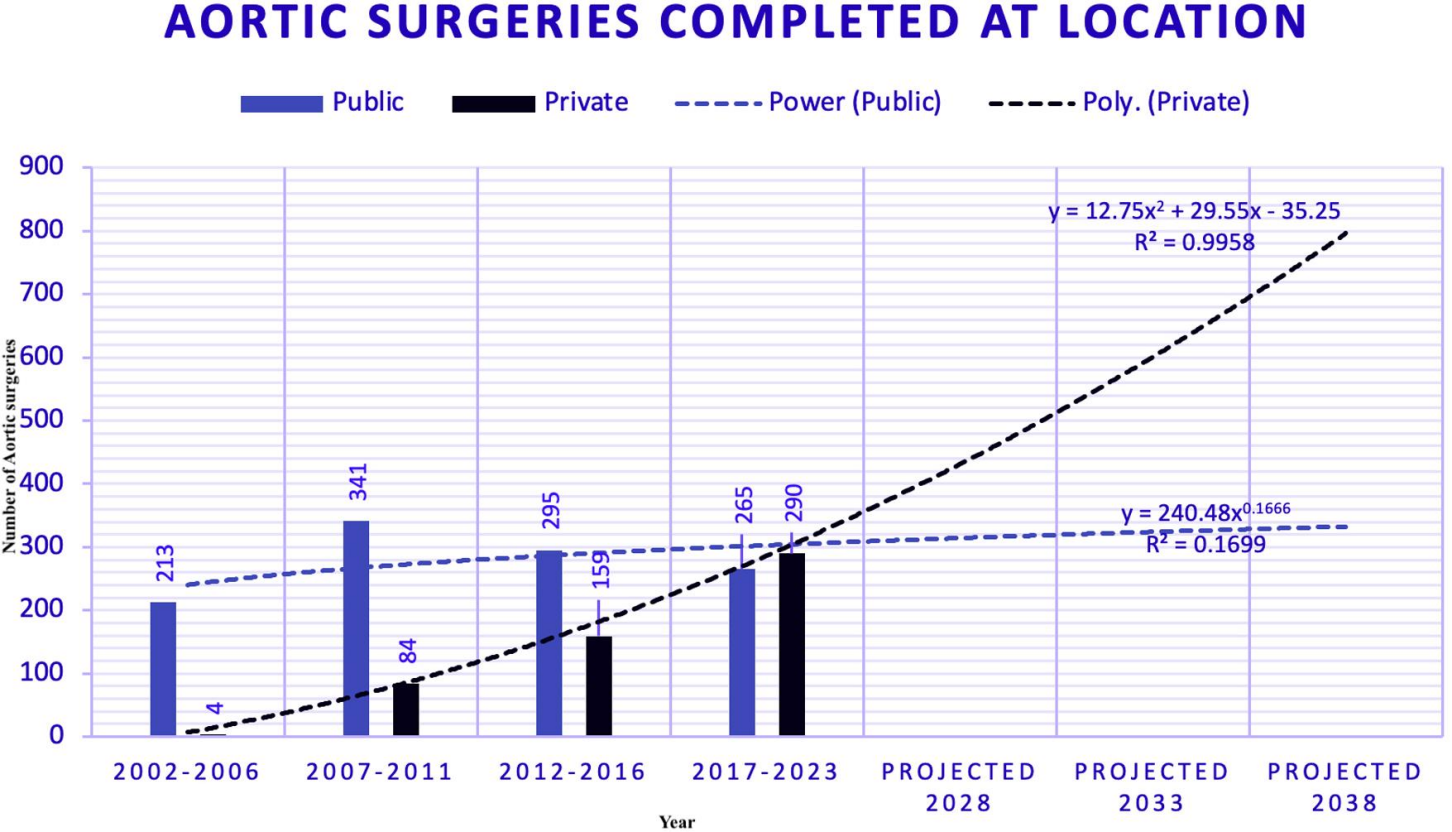
Aortic surgeries with trend models. A power model was chosen for public hospitals in this aortic dataset due to its highest R-squared value (0.17), indicating a modest fit. A polynomial model was selected for private hospitals, with an R-squared value of 0.99, reflecting a near-perfect fit. This discrepancy highlights the difference in predictability: public hospitals, with more variability and less managed services, have a lower prediction accuracy (17%), while private hospitals, with more consistent and managed services, achieve a prediction accuracy of 99%. Projections for the subsequent three periods show a significant divergence: by 2028, an estimated 314 surgeries in public hospitals and 431 in private hospitals; by 2038, these numbers are forecasted to reach 350 in public and 850 in private hospitals.

Trends in aortic surgeries across different locations, as estimated using polynomial regression —a subset of supervised machine learning —are displayed in Figure 5.

### Predicted carotid surgeries trends (2002–2023)

**Figure 6:**
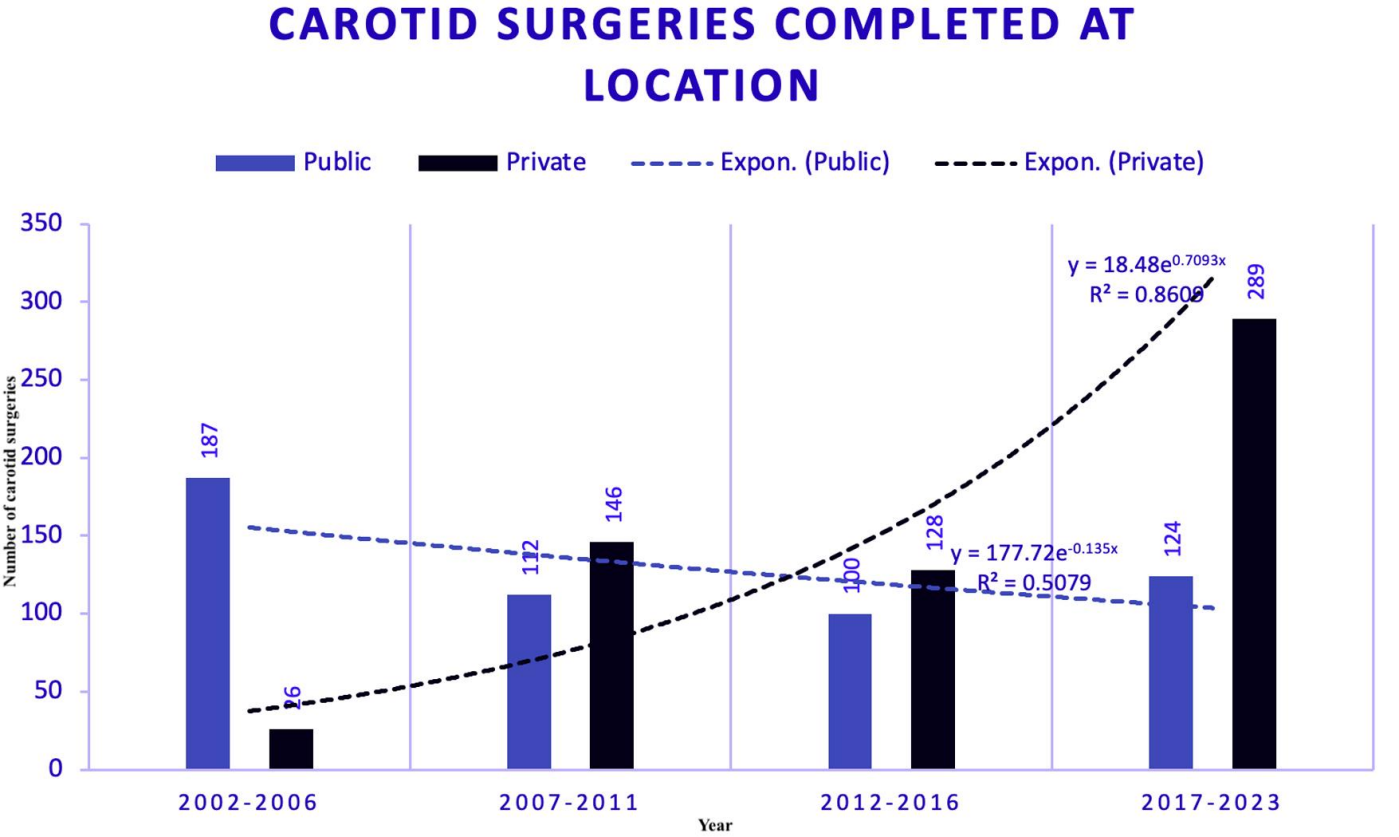
Carotid surgeries with trend models. Figure shows the trends in carotid surgeries at different locations using exponential modelling, a technique within supervised machine learning. For the carotid dataset, the Exponential Model was selected for both public and private hospitals due to its highest R-squared values, which are 0.51 for public hospitals and 0.86 for private hospitals. This indicates a moderate fit for public hospitals, reflecting some instability in trends, and a strong fit for private hospitals, indicating more apparent and reliable trends. Projections for 2028 estimate 90 surgeries in public hospitals and 641 in private hospitals, representing a 0.48-fold decrease in public compared to a 24-fold increase in private sectors. This stark contrast underscores the differing capacities and efficiencies between the two sectors.

Trends in carotid surgeries across different locations, as estimated using polynomial regression —a subset of supervised machine learning —are displayed in Figure 6.

### Predicted peripheral surgeries trends (2002–2023)

**Figure 7:**
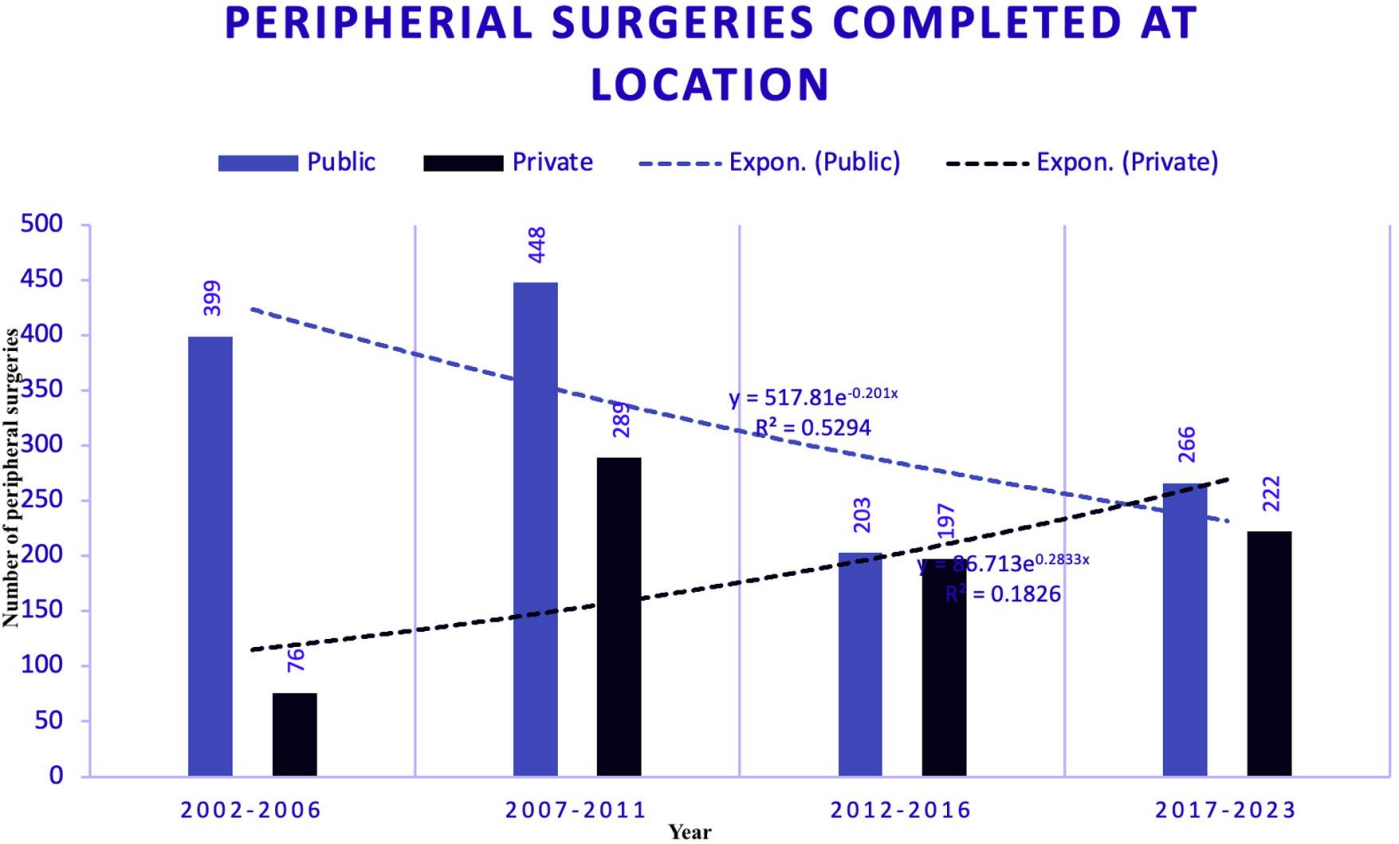
Peripheral surgeries with trend models. Figure illustrates the trends in peripheral surgeries at different locations using exponential modelling, a technique within supervised machine learning. For the peripheral dataset, the Exponential Model was chosen for public and private hospitals due to its highest R-squared values, which are 0.52 for public hospitals and 0.18 for private hospitals. The lower R-squared value for private hospitals indicates a less accurate model due to more dynamic and variable trends. Forecasts for 2028 estimate 189 surgeries in PUB and 357 in PRV. This suggests a notable difference in the growth rates and predictability between the two sectors.

Trends in peripheral surgeries across different locations, as estimated using polynomial regression —a subset of supervised machine learning —are displayed in Figure 7.

Figure seven illustrates the trends in peripheral surgeries at different locations using exponential modelling, a technique within supervised ML. For the peripheral dataset, the Exponential Model was chosen for public and private hospitals due to its highest R-squared values: 0.52 for public hospitals and 0.18 for private hospitals. The lower R-squared value for private hospitals indicates a less accurate model, likely due to more dynamic, variable trends. Forecasts for 2028 estimate 189 surgeries in PUB and 357 in PRV. This suggests a notable difference in the growth rates and predictability between the two sectors.

### Forecasts of aortic surgeries (2028–2053) (Figure 8)

Figure eight displays trend modelling techniques to forecast aortic surgeries by year. This chart shows that public hospitals operate under a capped model, indicating limited capacity for growth in surgical volumes. In contrast, private hospitals follow an uncapped model, showing a strong positive trend. This suggests that, assuming current conditions remain constant, private hospitals will continue to meet increasing demand without constraints.

**Figure 8:**
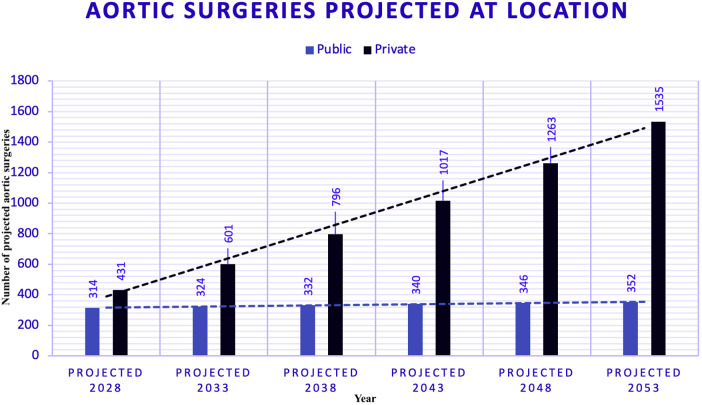
Forecasts of aortic surgeries - public (PUB) and private (PRV) hospitals. Figure displays trend modelling techniques to forecast aortic surgeries by year. This chart highlights that public hospitals follow a capped model, indicating a limited capacity for growth in surgical numbers. In contrast, private hospitals follow an uncapped model, showing a strong positive trend. This suggests that assuming current conditions remain constant, private hospitals will continue to meet increasing demand without constraints.

### Forecasts of carotid surgeries (2028–2038) (Figure 9)

Figure nine employs trend modelling techniques to forecast the number of carotid surgeries by year. This chart shows that public hospitals operate under a cap, suggesting limited capacity for growth in surgical volumes. In contrast, private hospitals follow an uncapped model, indicating a strong positive trend and the ability to meet increasing demand without restrictions.

**Figure 9:**
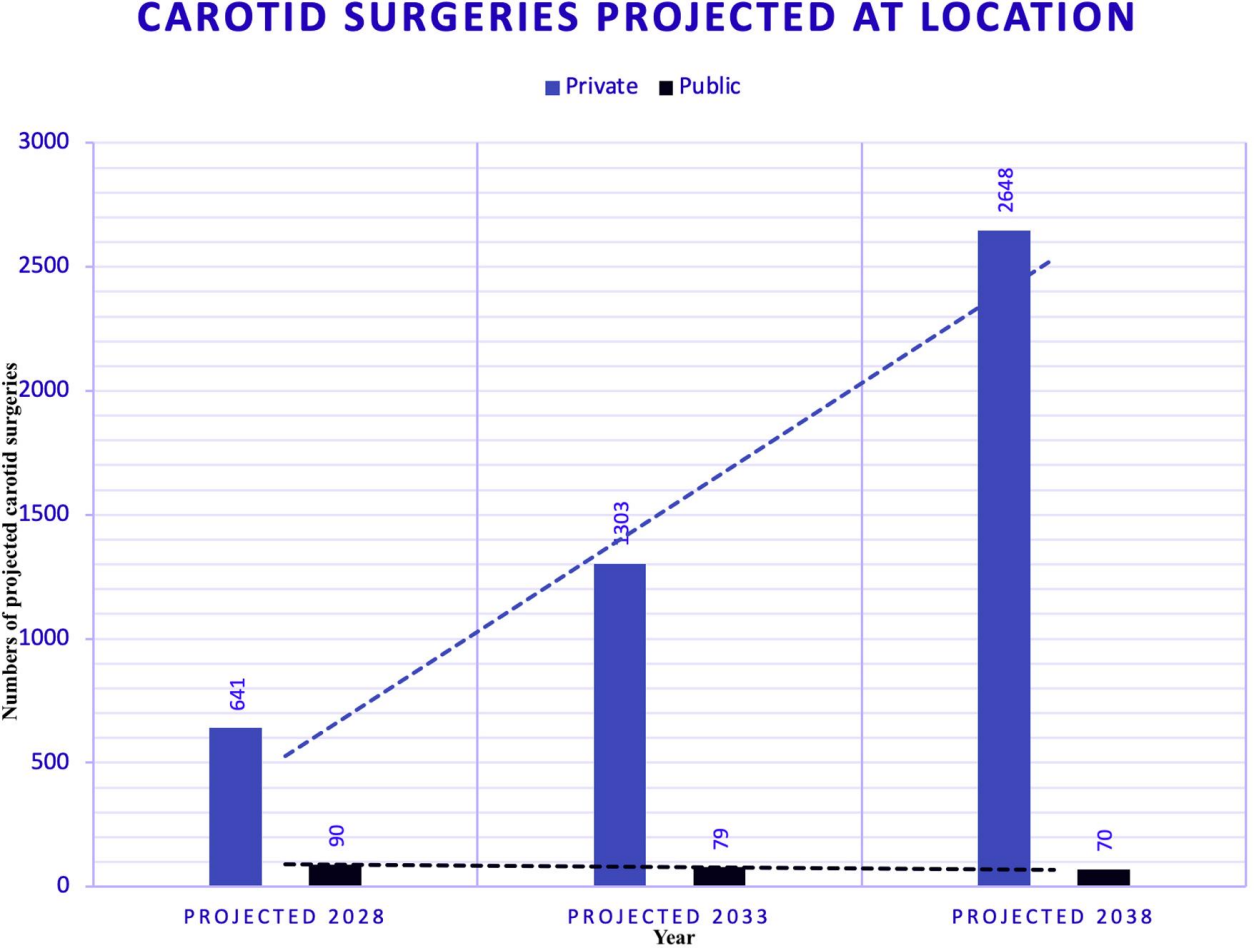
Forecasts of carotid surgeries - public (PUB) and private (PRV) hospitals. Figure employs trend modelling techniques to forecast carotid surgeries by year. This chart illustrates that public hospitals operate within a capped model, suggesting limited capacity for growth in surgical numbers. In contrast, private hospitals follow an uncapped model, indicating a strong positive trend and the ability to meet increasing demand without restrictions.

### Forecasts of peripheral surgeries (2028–2053) (Figure 10)

Figure ten uses trend modelling techniques to forecast peripheral surgeries by year. This chart shows that public hospitals operate under a capped model, suggesting limited capacity for growth in surgical volumes. In contrast, private hospitals follow an uncapped model, indicating a strong positive trend and the ability to meet increasing demand without restrictions.

**Figure 10:**
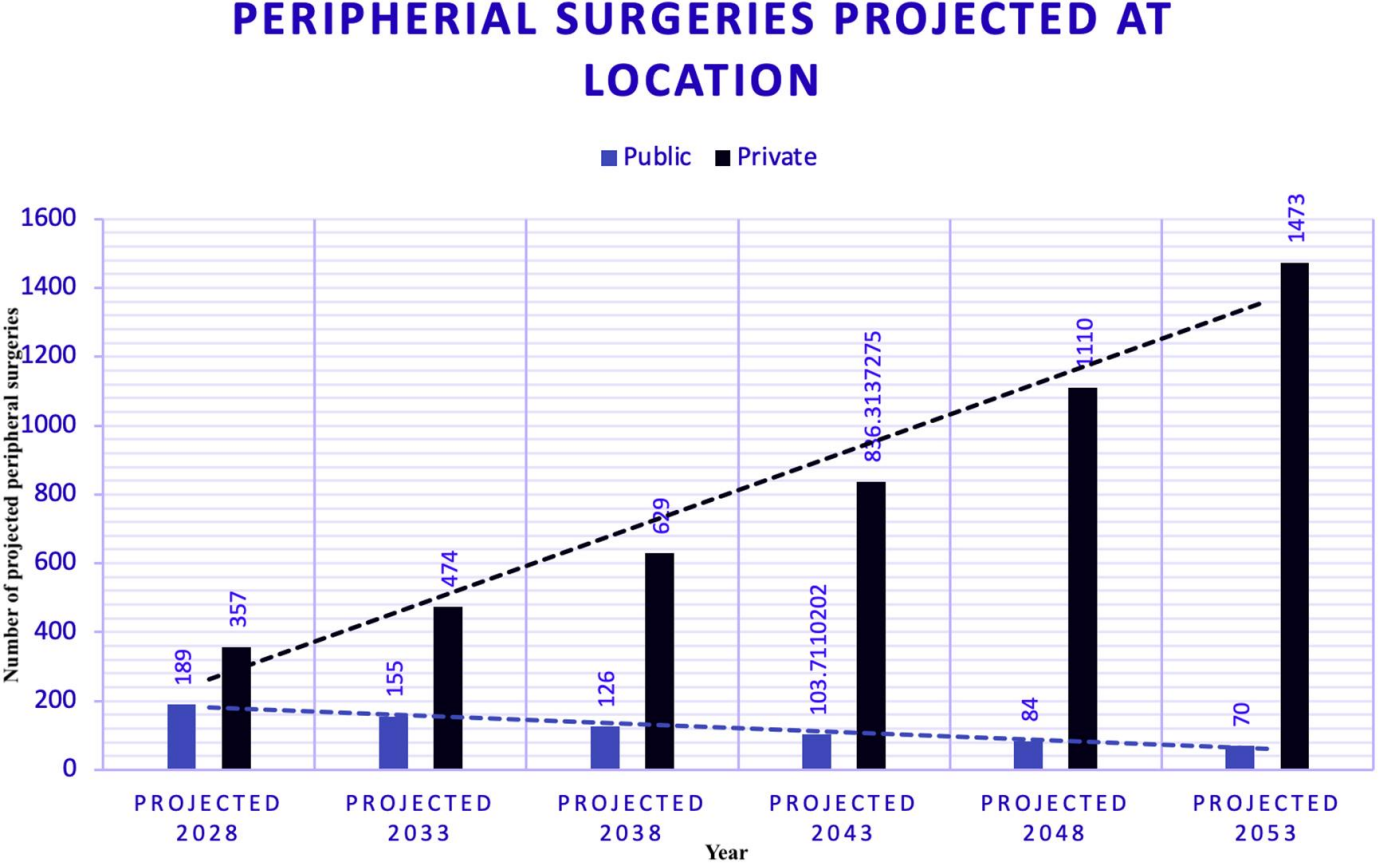
Forecasts of peripheral surgeries - public (PUB) and private (PRV) hospitals. Figure uses trend modelling techniques to forecast peripheral surgeries by year. This chart highlights that public hospitals follow a capped model, suggesting limited capacity for growth in surgical numbers. In contrast, private hospitals follow an uncapped model, indicating a strong positive trend and the ability to meet increasing demand without restrictions.

### Forecasts of size and capacity of vascular surgeons (2028–2053)

Currently, we have fewer than 50% of the required vascular surgeons working in Ireland. By 2030, the shortage of vascular surgeons in Ireland is projected to increase the clinical workload by 39%, requiring an additional 647,938 wRVUs, with a 28% workforce shortage. By 2050, shortages are expected to increase the clinical workload by 7%, necessitating an additional 727,585 wRVUs. Our model predicts a decline in surgeon shortages by 17% through 2040, with the workforce size meeting near demand by 2050, resulting in a 7% shortage (Table 1).

**Table 1:** Forecasting vascular surgery workforce shortage in Ireland by 2050.

Forecast^a^	2024	2030	2040	2050	2052
Projected population	5,089,478	5,457,058	5,825,548	6,127,854	6,380,401
Vascular Surgeons (VS) needed	71	76	82	86	89
Work relative value unit (wRVUs) needed	–	647,938	691,691	727,585	757,571
VSs forecasted	–	55	67	80	92
wRVUs forecasted	–	466,098	571,422	676,885	782,210
% Shortage	–	28%	17%	7%	−3%
The amount each future VS would need to increase production	–	39%	21%	7%	−3%
VSs forecasted	–	106.207	98.707	92.202	86.515

a Given values represent point estimates conditional on current assumptions about surgeon productivity, surgeon-to-population ratios, and census projections; they do not incorporate full statistical prediction intervals and should be interpreted as indicative ranges rather than exact forecasts.

## Discussion

In our use case, we analysed observed absolute numbers of vascular interventions in two large tertiary referral centres in the West of Ireland. We used data from 2002 to 2023 to examine trends and forecast future needs of healthcare personnel. We focused on the number of vascular surgeons' wRVUs until 2053. Our findings highlight the relative shortage of vascular surgeons and the need for better planning over the coming decades, including training for the future healthcare workforce (16). Our data show a declining shortage by 2040, similar to previous observations from Canada and the US (12). This is also supported by a recent study by Silvestre et al. (17) in US, which showed that vascular surgery workforce is projected to decrease significantly by 2037, especially in non-metropolitan areas. Although, we could not find Irish studies that forecasted vascular surgery workforce adequacy, previous studies in UK has forecasted a significant shortage of vascular surgeons in UK (18, 19).

However, there is scarcity of AI-based predictive workforce modelling. We believe through AI-based models, we can better predict workforce shortages and plan strategies to recruit and retain workforce (20). The observed trends and predictions highlight significant challenges and opportunities within the vascular surgery field in the West of Ireland. Our predictive modelling can be easily generalized to the rest of Ireland and worldwide, where the health care system is a hybrid of private and public services. Our results emphasize the striking differences in trends and predicted procedures between the public and private sectors, showing massive, linearly increasing differences in the number of forecasted vascular interventions by 2053 in private practice, vs. the fixed or declining number of interventions in public hospitals. This is more pronounced for peripheral vascular interventions (38-fold), Carotid interventions (21-fold), and aortic interventions (4.4-fold) in private vs. public settings. These differences are multifactorial but reflect Ireland's complex healthcare system and infrastructure, resulting in non-equitable healthcare provision to all citizens. Addressing healthcare disparities, promoting equitable access to advanced interventions, and sustaining a robust workforce should be the leading focus of policymakers and are paramount for the future of sustainable and equitable healthcare in Ireland. Our findings emphasize the clinical utility of leveraging AI tools and supervised ML for accurate forecasting and strategic planning. Our in-depth analyses could help navigate the complexities of workforce demands more effectively, ensuring optimal patient outcomes in the years ahead.

### Aortic surgery forecasting

We used a power model for PUB and a polynomial model for the PRV for aortic surgeries. The polynomial model at the PRV yielded an R-squared of 0.99, indicating a robust fit. In contrast, the power model for PUB had an R-squared of 0.17. The polynomial trend projects a significant increase in surgeries at the PRV, while the trend at PUB is relatively flat.

### Peripheral surgery forecasting

Peripheral surgeries at both locations were modelled using an exponential fit. The R-squared value for PUB was 0.53; for the PRV, it was lower but acceptable given the limited data. The trend at PUB shows a decline since 2007, whereas the PRV indicates a slow positive increase with a recent inflexion point, suggesting a shift in the trend.

### Carotid surgery forecasting

Carotid surgeries were also modelled using exponential trends. The private hospitals showed a strong positive trend (R-squared = 0.86), while the public hospitals displayed a flatter, decreasing trend (R-squared = 0.50). By 2028, private hospitals are expected to perform significantly more carotid surgeries than public hospitals.

The study predicts a shortage of vascular surgeons in the coming decades, with potential workforce size and demand alignment by 2050. Until then, vascular surgeons must increase their productivity to manage the workload. Burnout, changing practice patterns, geographic maldistribution, and expanding healthcare coverage will impact the future workforce's ability to meet population needs. Addressing future demands and capacity challenges through public-private partnerships, investing in vascular training, and improving wRVU compensation is crucial to ensure an adequate and sustainable vascular surgery workforce.

The rise of endovascular and hybrid interventions reflects the dynamic evolution toward individualized, multidisciplinary care models (16). Maintaining the current level of vascular practice requires a substantial increase in manpower. The growing demand for specialized interventions, such as aortic, carotid, and peripheral surgeries, requires recruiting and training more vascular surgeons with specific areas of expertise. Specialization allows surgeons to develop more profound proficiency and improve patient outcomes in their particular field, which is critical given the increasing complexity of vascular diseases (16).

Moreover, the increasing workloads and the projected need for higher productivity underline the importance of integrating AI and ML systems into vascular practice. These technologies can streamline administrative tasks, enhance diagnostic accuracy, and optimize surgical planning, freeing vascular surgeons to focus on complex interventions. By leveraging AI and ML, we can reduce strain on surgeons and improve efficiency across the healthcare system.

The reliance on wRVUs as a measure of productivity is unsustainable without significant changes in reimbursement policies. Current wRVU compensation models do not adequately reflect the increasing demands placed on vascular surgeons. To sustain the workforce and ensure high-quality care, there must be a 65% increase in reimbursement rates. This adjustment would compensate for the increased workload and help attract and retain talented surgeons (21).

### Study limitations

We were limited by the data, as we used only two vascular institutes within a geographical area for the predictive modelling. Furthermore, we used a subset of ML algorithms to improve model accuracy and forecast workforce needs, with accuracy assessed using R-squared values. The inherent limitations of having only four buckets of data over 22 years impact the accuracy of long-term predictions. Thus, our models prioritize the nearest buckets for more reliable forecasts, acknowledging that projections for 2023 are more accurate than those for 2050. Finally, although the two data sets from the public and private institutes exhibit different trends, we applied a single model to identify the model that best predicts future trends.

We did not perform explicit uncertainty quantification, as we used a basic supervised ML regression model in our time-series models, based on four aggregated, stratified time buckets over a 22-year period, across diverse datasets. Because the models are fitted on only four aggregated time periods, formal prediction intervals would not adequately capture structural uncertainty and would give a spurious impression of precision, particularly for forecasts beyond 2040. Our forecasts should therefore be interpreted as scenario-based estimates, with greater confidence in near-term projections and more caution for long-term horizons. Furthermore, R^2^ values should be interpreted appropriately to qualify the predictive strength of the model.

Furthermore, the benchmark of 1.4 vascular surgeons per 100,000 population was derived from U.S. workforce modelling, which may not be directly applicable to Ireland. Differences in population structure, the public–private mix, the distribution of vascular services, and the roles of interventional radiology and cardiology could mean that the optimal surgeon-to-population ratio for Ireland is higher or lower. Our projections should therefore be viewed as indicating the direction and scale of expected workforce strain rather than prescribing precise workforce targets for Ireland.

We used the full available dataset to estimate model parameters, given the limited number of time points and the exploratory nature of the analysis. No cross-validation was performed because there was a risk of “data leakage” in time-series data. Moreover, cross-validation methods might not properly handle non-independent grouped data.

## Conclusions

Our study shows significant disparities in healthcare access to vascular surgery between public and private practices. It is expected that the shortage of vascular surgeons will persist in both numbers and capacity over the coming decades. This could be addressed through strategic planning, increased specialization in vascular surgery and the integration of AI/ML systems into routine care to ensure adequate compensation and the sustainability of the workforce. A multicentre nationwide study is required to analyze trends in vascular surgeries across public and private institutions and to accurately forecast workforce requirements for future preparedness.

## Data Availability

The original contributions presented in the study are included in the article/Supplementary Material, further inquiries can be directed to the corresponding author.

## References

[1] AlHamzah M Hussain MA Greco E Zamzam A Jacob-Brassard J Wheatcroft M Trends in operative case volumes of Canadian vascular surgery trainees. J Vasc Surg. (2022) 75(2):687–694.e3. 10.1016/j.jvs.2021.07.230 34461218

[2] Hingorani A DerDerian T Gallagher J Ascher E. Recent trends in publications of US vascular surgery program directors. Vascular. (2014) 22(4):259–61. 10.1177/1708538113484464 23929419

[3] Jaff MR. Advances in the management of patients with vascular disease. Expert Rev Cardiovasc Ther. (2012) 10(2):151–3. 10.1586/erc.11.183 22292870

[4] Kirksey L. Health care disparity in the care of the vascular patient. Vasc Endovascular Surg. (2011) 45(5):418–21. 10.1177/1538574411407082 21527464

[5] Mendes Cde A Martins Ade A Teivelis MP Kuzniec S Wolosker N. Public private partnership in vascular surgery. Einstein (Sao Paulo). (2014) 12(3):342–6. 10.1590/s1679-45082014gs3029 25295457 PMC4872947

[6] Shalhub S Mouawad NJ Malgor RD Johnson AP Wohlauer MV Coogan SM Global vascular surgeons’ experience, stressors, and coping during the coronavirus disease 2019 pandemic. J Vasc Surg. (2021) 73(3):762–771.e4. 10.1016/j.jvs.2020.08.030 32882345 PMC7457940

[7] Martelli E Sotgiu G Saderi L Martelli AR Settembrini AM, Vascular Surgery Divisions of the Southern Regions of the Italian Peninsula. The impact of the first 11 months of the COVID-19 pandemic on vascular Patients’ care and hospitalisation rate in the vascular surgery divisions of southern Italy. Eur J Vasc Endovasc Surg. (2022) 64(2–3):274–5. 10.1016/j.ejvs.2022.04.021 35487389 PMC9040540

[8] Martelli E Sotgiu G Saderi L Federici M Sangiorgi G Zamboni M How the first year of the COVID-19 pandemic impacted Patients’ hospital admission and care in the vascular surgery divisions of the southern regions of the Italian Peninsula. J Pers Med. (2022) 12(7):1170. 10.3390/jpm12071170 35887667 PMC9316551

[9] Churpek MM Yuen TC Winslow C Meltzer DO Kattan MW Edelson DP. Multicenter comparison of machine learning methods and conventional regression for predicting clinical deterioration on the wards. Crit Care Med. (2016) 44(2):368–74. 10.1097/CCM.0000000000001571 26771782 PMC4736499

[10] Janse RJ Abu-Hanna A Vagliano I Stel VS Jager KJ Tripepi G When the whole is greater than the sum of its parts: why machine learning and conventional statistics are complementary for predicting future health outcomes. Clin Kidney J. (2025) 18(4):sfaf059. 10.1093/ckj/sfaf059 40276681 PMC12019231

[11] Population and Labour Force Projections 2023-2057 - Central Statistics Office. CSO (2024). Available online at: https://www.cso.ie/en/releasesandpublications/ep/p-plfp/populationandlabourforceprojections2023-2057/ (Accessed September 15, 2025).

[12] Go MR Oslock WM Way DP Baselice HE Tamer RM Kent KC An updated physician workforce model predicts a shortage of vascular surgeons for the next 20 years. Ann Vasc Surg. (2020) 66:282–8. 10.1016/j.avsg.2020.01.097 32027989

[13] Tuli S Hayes P O'Donoghue P Glynn F Scully R Murphy AW Politics, policy and action: lessons from rural GP advocacy in Ireland. Rural Remote Health. (2024) 24(4):8700. 10.22605/RRH8700 39563022

[14] Murray M Behan S Meegan S O'Brien W Smith C Goss H. Health literacy in Ireland: insights from rural and urban community perspectives. Health Promot Int. (2025) 40(3):daaf072. 10.1093/heapro/daaf072 40498772 PMC12154202

[15] Johnston BM Burke S Barry S Normand C Ní Fhallúin M Thomas S. Private health expenditure in Ireland: assessing the affordability of private financing of health care. Health Policy. (2019) 123(10):963–9. 10.1016/j.healthpol.2019.08.002 31421910

[16] Jayroe H Weaver L Velazquez G Nelson P Jennings W Henning N Vascular surgery training positions and applicant 10-year trends with consideration for further expansion. Ann Vasc Surg. (2023) 95:291–6. 10.1016/j.avsg.2023.05.003 37247836

[17] Silvestre J Wooster MD Seeger S Rowe VL Reitman CA. Trends in supply, demand, and workforce adequacy in vascular surgery: forecasting a national shortage. J Vasc Surg. (2025) 82(3):1066–72. 10.1016/j.jvs.2025.04.071 40449700

[18] Harkin DW Beard JD Shearman CP Wyatt MG. The vascular surgery workforce: a survey of consultant vascular surgeons in the UK, 2014. Eur J Vasc Endovasc Surg. (2015) 49(4):448–54. 10.1016/j.ejvs.2014.11.008 25544313

[19] Harkin DW Beard JD Shearman CP Wyatt MG. Predicted shortage of vascular surgeons in the United Kingdom: a matter for debate? Surgeon. (2016) 14(5):245–51. 10.1016/j.surge.2015.10.004 26654693

[20] Bositkhanova N Dadaboyev SMU. Revolutionizing workforce planning: the strategic role of AI in HR strategy. Discov Glob Soc. (2025) 3(1):100. 10.1007/s44282-025-00252-y

[21] Satiani B. Use, misuse, and underuse of work relative value units in a vascular surgery practice. J Vasc Surg. (2012) 56(1):267–72. 10.1016/j.jvs.2012.03.013 22579074

