# Supplementary material for: Predictive modelling of vascular surgery trends using machine learning: a comparative study of Irish public and private tertiary referral centres
Source: Front Surg. 2026 Jan 8;12:1733205. doi: 10.3389/fsurg.2025.1733205 (PMC12823813; doi:10.3389/fsurg.2025.1733205)
Supplement: Supplementary file 1 [file Supplementaryfile1.docx]

### Supplementary Table 1. Future vascular surgeons' workforce needs and predictions (estimated projection based on 1.2 vascular surgeons needed per 100,000 population the United States).

| **United States** | **2017** | **2020** | **2030** | **2040** | **2050** |
| --- | --- | --- | --- | --- | --- |
| **Projected population** | 310,233,000 | 332,639,102 | 355,100,730 | 373,527,973 | 388,922,201 |
| **Vascular Surgeons (VS) needed** |  | 3992 | 4261 | 4482 | 4667 |
| **Work relative value unit (wRVUs) needed** |  | 33,853,346.69 | 36,139,311.49 | 38,014,688.87 | 39,581,390.24 |
| **VSs forecasted** | 3232 | 3350 | 4107 | 4865 | 5622 |
| **wRVUs forecasted** |  | 28,411,350 | 34,831,467 | 41,260,065 | 47,680,182 |
| **% Shortage** |  | 16% | 4% | -9% | -20% |
| **Amount each future VS would need to increase production** |  | 19% | 4% | -8% | -17% |
| **Ireland** | **2024** | **2030** | **2040** | **2050** | **20502** |
| **Projected population** | 5,089,478 | 5,457,058 | 5,825,548 | 6,127,854 | 6,380,401 |
| **VSs needed** |  | 65 | 70 | 74 | 77 |
| **wRVUs needed** |  | 555,376 | 592,878 | 623,644 | 649,346 |
| **VSs forecasted** |  | 55 | 67 | 80 | 92 |
| **wRVUs forecasted** |  | 466,098 | 571,422 | 676,885 | 782,210 |
| **% Shortage** |  | 16% | 4% | -9% | -20% |
| **Amount each future VS would need to increase production** |  | 19% | 4% | -8% | -17% |
| **VSs forecasted** |  | 78.028 | 72.531 | 67.750 | 63.560 |

### Supplementary Table 2. Future vascular surgeons' workforce needs and predictions (estimated projection based on 1.4 vascular surgeons per 100,000 population in the United States).

| **United States** | **2017** | **2020** | **2030** | **2040** | **2050** |
| --- | --- | --- | --- | --- | --- |
| **Projected population** | 310233000 | 332639102 | 355100730 | 373527973 | 388922201 |
| **Vascular Surgeons (VS) needed** |  | 4,657 | 4,971 | 5,229 | 5,445 |
| **Work relative value unit (wRVUs) needed** |  | 39495571 | 42162530 | 44350470 | 46178289 |
| **VSs forecasted** | 3232 | 3,350.00 | 4,107.00 | 4,865.00 | 5,622.00 |
| **wRVUs forecasted** |  | 28411350 | 34831467 | 41260065 | 47680182 |
| **% Shortage** |  | 0 | 0 | 0 | 0 |
| **Amount each future VS would need to increase production** |  | 39% | 21% | 7% | -3% |
| **VSs forecasted** |  | 328300% | 402500% | 476800% | 551000% |
| **Ireland** | **2024** | **2030** | **2040** | **2050** | **20502** |
| **Projected population** | 5089478 | 5457057.733 | 5825548.388 | 6127853.584 | 6380401.136 |
| **VSs needed** |  | 76 | 82 | 86 | 89 |
| **wRVUs needed** |  | 647938 | 691691 | 727585 | 757571 |
| **VSs forecasted** |  | 55 | 67 | 80 | 92 |
| **wRVUs forecasted** |  | 466098 | 571422 | 676885 | 782210 |
| **% Shortage** |  | 28% | 17% | 7% | -3% |
| **Amount each future VS would need to increase production** |  | 39% | 21% | 7% | -3% |
| **VSs forecasted** |  | 106.207 | 98.707 | 92.202 | 86.515 |

### Supplementary Table 3. Future vascular surgeons' workforce needs and predictions (estimated projection based on 1.6 vascular surgeons needed per 100,000 population the United States).

| **United States** | **2017** | **2020** | **2030** | **2040** | **2050** |
| --- | --- | --- | --- | --- | --- |
| **Projected population** | 310,233,000 | 332,639,102 | 355,100,730 | 373,527,973 | 388,922,201 |
| **Vascular Surgeons (VS) needed** |  | 5322 | 5682 | 5976 | 6223 |
| **Work relative value unit (wRVUs) needed** |  | 45,137,795.58 | 48,185,748.66 | 50,686,251.82 | 52,775,186.99 |
| **VSs forecasted** | 3232 | 3350 | 4107 | 4865 | 5622 |
| **wRVUs forecasted** |  | 28,411,350 | 34,831,467 | 41,260,065 | 47,680,182 |
| **% Shortage** |  | 37% | 28% | 19% | 10% |
| **Amount each future VS would need to increase production** |  | 59% | 38% | 23% | 11% |
| **Ireland** | **2024** | **2030** | **2040** | **2050** | **20502** |
| **Projected population** | 5,089,478 | 5,457,058 | 5,825,548 | 6,127,854 | 6,380,401 |
| **VSs needed** |  | 87 | 93 | 98 | 102 |
| **wRVUs needed** |  | 740,501 | 790,504 | 831,525 | 865,795 |
| **VSs forecasted** |  | 55 | 67 | 80 | 92 |
| **wRVUs forecasted** |  | 466,098 | 571,422 | 676,885 | 782,210 |
| **% Shortage** |  | 37% | 28% | 19% | 10% |
| **Amount each future VS would need to increase production** |  | 59% | 38% | 23% | 11% |
| **VSs forecasted** |  | 138.716 | 128.945 | 120.445 | 112.995 |
